# Doping density, not valency, influences catalytic metal-assisted plasma etching of silicon†

**DOI:** 10.1039/d3mh00649b

**Published:** 2023-06-19

**Authors:** Julia B Sun, Namphung Peimyoo, James O Douglas, Benjamin D Almquist

**Affiliations:** a Department of Bioengineering, Imperial College London, Royal School of Mines Exhibition Road London SW7 2AZ UK b.almquist@imperial.ac.uk; b Department of Materials, Imperial College London, Royal School of Mines Exhibition Road London SW7 2AZ UK

## Abstract

Metal-assisted plasma etching (MAPE) of silicon (Si) is an etching technique driven by the catalytic activity of metals such as gold in fluorine-based plasma environments. In this work, the role of the Si substrate was investigated by examining the effects of the dopant concentration in both n- and p-type Si and the dopant atom type in n-type Si in SF_6_/O_2_ mixed gas plasma. At the highest dopant concentrations, both n- and p-type Si initially exhibit inhibition of the MAPE-enhanced etching. As the etch progresses, MAPE initiates, resulting in catalytic etching of the underlying Si at the metal–Si interface. Interestingly, MAPE-enhanced etching increases with decreasing doping concentrations for both n- and p-type Si substrates, distinct from results for the similar but divergent, metal-assisted chemical etching of silicon in liquid. Our findings show that the metal–Si interface remains essential to MAPE, and surface enrichment of the dopant atoms or other surface chemistries and the size of metal nanoparticles play roles in modulating catalytic activity.

## Abstract 2

Gold catalyses Si etching in dry plasmas *via* Metal-Assisted Plasma Etching (MAPE). Here, MAPE is shown to be uniquely inhibited by both heavily doped n- and p-type Si, in contrast with reactive ion etching and metal assisted chemical etching (MACE).

## Graphical abstract

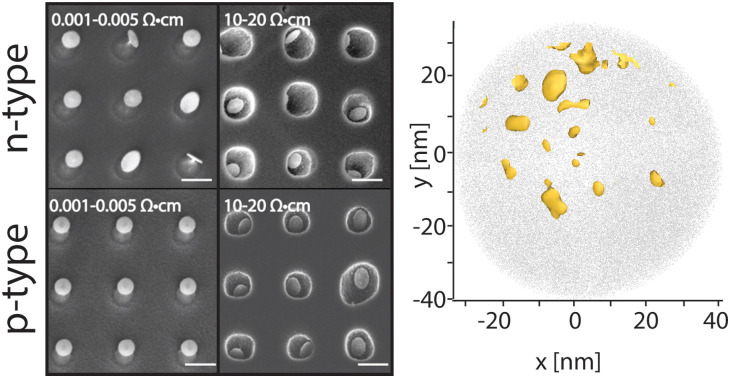

**New concepts**

Over 40 years ago, the mechanism underpinning how metals like gold enhance the etching of Si in dry plasmas was mischaracterised. This incorrect explanation stood for decades until recently, when new research demonstrated that a different mechanism, metal-assisted plasma etching (MAPE), which relies on direct interfacial contact between the metal and silicon, underpins this phenomenon. Intriguingly, the process of MAPE fundamentally differs from the process of metal-assisted chemical etching (MACE) during wet etching. This is fascinating since both processes harness metal–silicon contact in the presence of fluorine-based, oxidising etchants to drive enhanced etching. Here, we find the surprising result that MAPE is inhibited in both highly doped n- and p-type silicon. This directly contradicts MACE, reactive ion etching (RIE), and other etching methods based on chemical reactions. In these, n-type silicon facilitates enhanced etching, while p-type silicon reduces the etch rate. Taken together, this work demonstrates that MAPE is truly a unique phenomenon since it is affected by the concentration, not the polarity, of the dopant atom, despite being a reaction-based, not physical, etch. This is intriguing since all previous mechanisms proposed for the dopant-dependent etching seen during MACE do not apply to MAPE. We further characterise this process using atom probe tomography to elucidate new details on the role of the catalytic gold during the etching process, which sheds light on how it may be possible to begin to control and harness this mechanism.

## 1. Introduction

Fabrication of patterned and controllable Si nanostructures are of great importance for a broad range of applications, including nanoelectronics,^1^ optoelectronics,^1^ energy storage,^2^ bio- and chemical sensors^3,4^ and bioimaging.^5^ Various techniques for fabricating Si nanostructures have been developed, such as metal-assisted chemical etching (MACE) in liquids and dry etching with plasmas. In reactive ion etching (RIE), fluorine-containing plasma, *i.e.* SF_6_ and CF_4_, are commonly used for silicon processing. Compared to wet etching, dry etching with plasma offers more isotropic control, precision, and higher etch rates to achieve high-aspect-ratio nanostructures.

Metallic masks such as Au, Ag, Cu, and Al demonstrated the ability to generally increase the etch rate of Si and SiO_2_ in fluorine-based gases.^6–8^ Previously, we demonstrated the existence of metal-assisted plasma etching (MAPE) – the local, catalytic enhancement of Si etching in plasma due to direct interfacial contact with metallic masks, leading to the etch rate increasing up to 1000%.^9^ Due to the requirement for Si–metal contact, this enhancement in etching localises in the region of the catalytic metal, not as a global increase in Si etch rate. Interestingly, we found that the mechanism underpinning the enhancement in etching during dry MAPE diverges from traditional solution-based MACE, which is governed by electrochemical and mass transport reactions.^10^ Molecular dynamics simulations have shown that during chemical etching of silicon, the higher electronegativity of Au than Si attracts electron density away from Si, creating active etching sites for electron-rich oxidising agents.^11^ However, electronegativity governing charge transfer between metal and Si is limited in explaining the MACE process for some metal catalysts.^12^ In the gas phase, numerous simulations have explored various etching reactions of Si in fluorine-based gases,^13–15^ but to our knowledge, there are no computational studies on the adsorption mechanism of reactive ions and radicals on Au surface during dry etching of Si that may shed light on the role of Au during MAPE.

While our previous work established the existence of MAPE, much is still unknown regarding this process. For instance, the level of doping in Si significantly influences etch rate in both traditional dry and wet etching environments. For polycrystal Si substrates etched in halogen-based plasmas, heavily doped n-type Si etches faster than undoped Si, which in turn etches faster than heavily doped p-type Si.^16–18^ The possible mechanism for this differential behaviour is the opposite polarity of the depletion region created by Fermi-level pinning at the Si surface.^17^ In n-type polySi, Coulombic attraction between the donor dopant and negative halogen atoms pulls halogen atoms into the Si lattice, increasing the etch rate. In contrast, for p-type Si, Coulombic repulsion occurs, driving a decrease in the etch rate.

In MACE, Si etch rates and morphologies also depend on the doping concentration and dopant type. Previous MACE studies using Ag catalysts with various doping levels of p-type (B dopant) and n-type (P dopant) Si wafers showed that heavily doped n-type Si exhibited higher etch rates than lightly doped n- and p-type Si, which etched faster than heavily doped p-type Si,^19^ in agreement with the results for plasma etching. Similarly, the MACE system for Au catalytic films showed n-type Si is etched faster than p-type Si.^20^ Specifically, the highly doped p-type Si substrate etches significantly less than the moderately and lightly doped substrates. Additional to the etch rate, the doping level in Si also affects the morphology of the etched structures.^10^ As the doping level increases, Si nanowires formed by MACE become more porous.^21,22^ One possible cause is the presence of more crystal defects or impurities in higher dopant samples acting as nucleation sites for pore formation.^23^ Another proposed mechanism suggests this is due to less band bending at the metal–semiconductor interface in highly doped Si compared to the lightly doped samples,^19^ leading to more efficient diffusion of holes to Si regions and resulting in increased roughness and porosity. All in all, though, the mechanism underpinning the influence of doping levels on etch rates during MACE is not fully understood.

With that being said, the etching process of MAPE fundamentally differs from that of MACE, as plasma etching proceeds *via* both physical and chemical reactions.^9^ To the best of our knowledge, the influence of doping concentration on MAPE in Si is unknown. In this work, we explore how catalytic etching in MAPE is influenced by the concentration and type of dopants in the Si substrates, aiming to further understand the mechanism underpinning the MAPE process.

## 2. Experimental section

### 2.1. Semiconductor substrates

Single-crystal silicon wafers with different resistivities, doping levels, dopant types were obtained from Inseto UK Limited and University Wafers, USA. Substrate properties of the semiconductor wafers used in this study are summarised in Table 1. Before processing, all semiconductor substrates were cleaned in successive acetone and isopropanol rinses and dried with N_2_ gas.

**Table 1:** Properties of semiconductor wafers

Material	Type	Doping	Surface orientation	Resistivity (Ω cm)	Dopant concentration (atoms per cm^3^)
Si	n-Type	Phosphorus	(100)	0.001–0.005	1 × 10^19^–8 × 10^19 ^a
				0.08–0.5	1 × 10^16^–1 × 10^17 ^a
				1–10	3 × 10^14^–4 × 10^15^
				10–20	2 × 10^14^–3 × 10^14^

Si	p-Type	Boron	(100)	0.001–0.005	3 × 10^19^–1 × 10^20^
				0.005–0.1	3 × 10^17^–2 × 10^19^^a^
				1–10	1 × 10^15^–1 × 10^16^
				10–20	7 × 10^14^–1 × 10^15^

Si	Undoped	Undoped	(100)	>10 000	Not specified

Si	n-Type	Arsenic	(100)	0.001–0.005	1 × 10^19^–7 × 10^19^
		Antimony	(100)	0.003–0.004	1 × 10^19^–2 × 10^19^

a Not provided by manufacturer. Dopant concentration is approximated using the PV lighthouse resistivity calculator at *T* = 300 K.^24^

### 2.2. Fabrication of thin film nanoparticle arrays

Cleaned semiconductor substrates were spin-coated with 100 nm of PMMA (Microchem, USA, 950 K molecular weight) and baked at 160 °C for 2 min. Nanoparticles measuring approximately 200 nm in diameter were patterned using the Elionix ELS-G100 100 kV EBL system (Eliniox Inc., Japan) and developed in a 1 : 3 mixture of methyl isobutyl ketone in isopropanol. Resist descum was performed in O_2_ plasma for 2 min in a Diener plasma generator (Diener electronic GmbH & Co. KG, Germany). Thin films of 5 nm Au and 10 nm Cr were deposited using an Edwards A500-FL500 electron beam metal evaporator (Edwards High Vacuum International, UK). After overnight lift-off in MICROPOSIT Remover 1165 solvent (Dow Electronic Materials, USA), the patterned nanoparticle arrays were ready for MAPE processing.

### 2.3. Metal-assisted plasma etching

Metal-assisted plasma etching of patterned semiconductor substrates was performed by mixed gas SF_6_/O_2_ plasma etching in the PlasmaPro NGP80 plasma processing system (Oxford Instruments, UK). MAPE was carried out at a base pressure of 175 mTorr and RF power of 50 W. The gas flow rate was kept constant with 30 sccm SF_6_ and 10 sccm O_2_. The time of etch was varied between 1 to 3 min.

### 2.4. SEM imaging

MAPE-treated semiconductor substrates were visualised by top-view and tilting SEM imaging in a Zeiss XB1540 (Carl Zeiss AG, Germany). Tilting SEM imaging was executed at a 45° tilt.

### 2.5. Atom probe tomography

Before focused ion beam (FIB) sample preparation, the prepared surfaces were coated with a capping layer of chromium by electron beam evaporation with thickness of 150 nm. Next, Pt lines and a main protective Pt layer were deposited by electron beam and FIB deposition. FIB lift-out and FIB sharpening^25^ of the APT specimens were performed on a Helios 5 DualBeam (Thermofisher Scientific) and a Helios 5 PFIB (Thermofisher Scientific) with a 5 kV low energy polish of Ga ions or Xe ions, respectively. Annular milling enabled sharpening of the APT specimens to a final tip diameter of less than 100 nm. Atom probe analysis was carried out on a Cameca Local Electrode Atom Probe (LEAP) 5000 XR at a base temperature of 50 K, a base vacuum less than 6 × 10^−11^ torr, and a variable pulse rate between 100–150 kHz such that Au^+^ ions at 197 Da could always be detected.

## 3. Results & discussion

### Effect of silicon doping level in MAPE

**Fig. 1:**
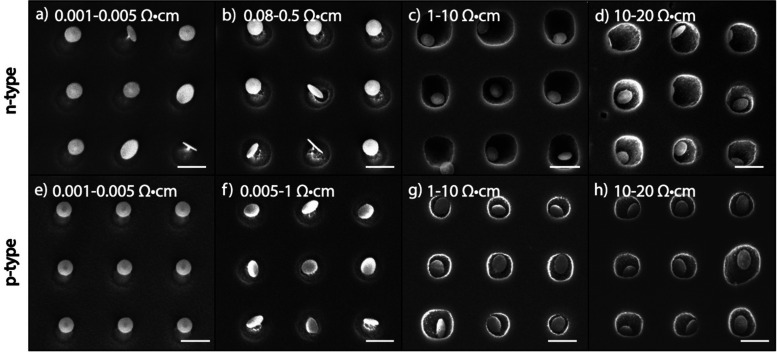
MAPE enhanced etching of n- and p-type Si substrates with various doping concentration. Silicon substrates were patterned with 5 nm Au/10 nm Cr nanoparticles and etched for 1 min in SF_6_/O_2_ at 25% O_2_ concentration. (a) Heavily doped n-type Si (resistivity 0.001–0.005 Ω cm). (b) Less heavily doped n-type Si substrates (resistivity 0.08–0.5 Ω cm). (c and d) Lightly doped n-type Si with resistivities of 1–10 and 10–20 Ω cm, respectively. (e) Heavily doped p-type Si with resistivity of 0.001–0.005 Ω cm. (f) The less heavily doped p-type substrates (resistivity 0.005–1 Ω cm). (g and h) Lightly doped p-type Si with resistivities of 1–10 and 10–20 Ω cm, respectively. All scale bars represent 500 nm.

In order to examine the effect of substrate doping in MAPE, n-type Si substrates doped with phosphorus (P) and p-type Si substrates doped with boron (B) were patterned with 5 nm Au/10 nm Cr nanoparticle arrays and etched for one min in SF_6_/O_2_ mixed plasma (Fig. 1). The ranges of doping concentrations used in this study are shown in Table 1. For both n-and p-type Si, the heaviest doped substrates measure 0.001–0.005 Ω cm in resistivity, which corresponds to approximate dopant concentrations of 10^19^–10^20^ atoms per cm^3^, and the lightest doped substrates measure 10–20 Ω cm in resistivity, corresponding to dopant concentrations of 10^14^–10^15^ atoms per cm^3^.

Significant differences are seen in the variously doped silicon substrates after 1 min of etching with MAPE. In the more heavily doped n-type silicon substrates (resistivity <0.005 Ω cm), there appears to be an inhibition of MAPE, wherein silicon pillars form instead of the familiar pitting profiles in MAPE-enhanced etching^9^ (Fig. 1a and b). The formation of the pillars is a typical isotropic etching characteristic in SF_6_-based gas chemistries and indicates that the Au/Cr patterned nanoparticles are acting as etch masks instead of MAPE catalysts. As the concentration of dopant atoms decreases, the MAPE inhibition effect seemingly diminishes and the enhanced catalytic effect in MAPE is reestablished (Fig. 1c and d). This is visualised by the collapse of the silicon pillars in favour of the formation of the etch pits. The etch profiles observed in lightly doped n-type silicon substrates (resistivities 1–10 Ω cm, 10–20 Ω cm) resemble our previous report on Au-catalysed Si etch at 1 min in the MAPE environment.^9^ Interestingly, the same pattern of inhibition at high dopant concentrations and catalytic etching at low dopant concentrations is also exhibited in p-type Si substrates (Fig. 1e–h). Clearly, it is shown in both n- and p- type Si substrates etched for 1 min that higher levels of doping seemingly correlate with greater inhibition of catalytic etching, in contrast to etching Si by both RIE and MACE where heavy n- and p- doping display opposite behaviours (fast *versus* slow etching, respectively).

As the Si etch time increases to 2 and 3 min, the inhibition of etching in heavily doped Si substrates is lost for both n- and p-type Si, and no major differences in the MAPE-catalysed etching effect are observed across doping densities (Fig. S1 and S2, ESI†). At 2 min, the nanoparticles exhibit a more controlled etch of the underlying Si, creating well-ordered etched arrays corresponding to the position of each original nanoparticle catalyst (Fig. S1e–h and S2e–h, ESI†). As the etch extends to 3 min, this effect transforms into the more disordered etched pits as reported in our previous work^9^ (Fig. S1i–l and S2i–l, ESI†). The disordered etching is likely due to stochastic movement of the nanoparticle catalysts in the plasma environment, which disrupts the initial lattice features at 2 min and forms less uniform pits as the etch progresses. The catalytic etching effect does not significantly extend beyond the boundaries of the Au–Si interface, denoting that any enhancement in etch is localised to the nanoparticle arrays and is not an overall enhancement of the etch environment. For both heavily and lightly doped n- and p-type Si substrates, MAPE-catalysed etching beyond 1 min seems to proceed independently of the level of substrate doping, and any dopant-dependent initial inhibition effects do not persist as the etch advances.

The early differences observed in MAPE etching appear between substrates of different doping levels and do not differ between n- and p-type substrates with the same doping levels. The inhibition effect is only observed in heavily doped substrates, irrespective of n- or p-type classification, and only at the earliest etching time point. To further confirm the hypothesis that the doping level affects MAPE inhibition, undoped Si substrates (resistivity >10 000 Ω cm) were etched for 1, 2, and 3 min in SF_6_/O_2_ mixed plasma. The etch proceeded in the familiar MAPE-catalysed manner for all three etch times, similar to the lightly doped n- and p-type Si substrates (Fig. S3, ESI†). This leads to the conclusion that the higher concentration of dopant atoms inhibits the MAPE process in Si substrates, and this inhibition of MAPE etching is not affected by whether the dopant is supravalent or subvalent for substrates of the same dopant level.

### Effect of the dopant atom type

**Fig. 2:**
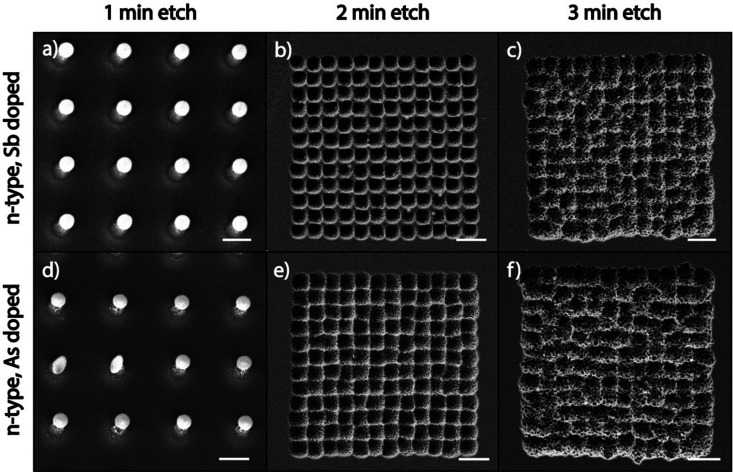
MAPE etching of heavily Sb and As doped n-type Si substrates. (a–c) SEM images of n-type Si substrates with Sb dopants (resistivity 0.003–0.004 Ω cm) patterned with Au/Cr circular nanoparticles by electron beam lithography and etched in SF_6_/O_2_ mixed plasma at 25% O_2_ concentration for 1, 2, 3 min, respectively. (d–f) SEM images of n-type Si doped with As (resistivity 0.001–0.005 Ω cm) prepared and etched under the same conditions as Sb-doped samples for 1, 2 and 3 min, respectively, exhibiting similar inhibitory and catalytically enhanced etching characteristics as in the Sb-doped Si substrates. Note that for 2 and 3 min etch time, the figures show pitting features in the shapes of the 12 × 12 array. Scale bars in panels a and d represent 500 nm, and all other scale bars represent 2 μm.

Since high dopant concentrations in the substrate inhibit the catalytic etching of MAPE, we next explored whether the type of dopant atom modulates the ability of highly doped Si substrates to inhibit MAPE. Fig. 2 shows SEM images of Au/Cr arrays patterned on highly doped n-type Si substrates, which are doped with antimony (Sb) and arsenic (As) after etching in the MAPE environment for 1, 2, and 3 min. Inhibition of the MAPE-catalysed etching is seen in all three highly doped substrates following 1 min of etching, as evidenced by Si pillars underneath each nanoparticle (Fig. 2a and d). As the etching progresses, the familiar MAPE-catalysed pitting reappears in the 2 min etch (Fig. 2b and e) and develops into the more stochastic etched features by 3 min (Fig. 2c and f). However, the etch pitting remains within the origin array boundaries. Both the Sb- and As-doped substrates do not continue to sustain the inhibitory effect as the etching advances in time, similar to the P- and B-doped substrates (Fig. S1 and S2, ESI†). These results indicate that the inhibition of MAPE at high dopant concentrations holds true for all heavily doped Si substrates tested in this study, regardless of the period of the dopant atom. The inhibition of MAPE in heavily doped Si is likely caused by the increased concentration of the dopant atoms and not by interactions between a specific type of dopant atom and the Si crystal lattice, confirming that the type of dopant atom does not affect the enhanced catalytic etching effect at the Au–Si interface during MAPE.

### Doping dependency in MACE and MAPE

To try to understand why heavily doped substrates exhibit inhibition during MAPE etching, we can compare the data from this study to the doping dependency in the analogous wet etching method, MACE. The difference in etching profile and etch rates as a function of the level of doping is well known in MACE. In MACE, two competing processes determine the etch features and etch rate in highly doped Si: (1) the characteristic metal-catalysed vertical etching, and (2) porosification – the introduction of additional etching pathways due to increased availability of electronic holes at higher doping levels.^26^ This porosification occurs across the Si substrate and causes unintended etching in non-metallised areas. Overall, MACE etching creates greater etch depths in highly doped n-type Si than lightly doped n- and p-type samples, which in turn exhibit greater etch depths than highly doped p-type Si.^27^ Because of the irregularity in etch depth and rate in MACE processing depending on the Si doping, the concentration of the MACE etchants must be adjusted to accommodate for the resistivity and doping type of the Si substrates to achieve uniform etching.^26,28–30^

In MACE, several proposed explanations exist for the differences in etch porosity and etch rate at high doping levels. Higher doping concentrations increase the amount of weak defective sites in the Si lattice, which is proposed to initiate the formation of additional etching pathways.^31^ In MACE for Au catalysis, highly doped n-type Si etches faster than highly doped p-type Si, while the etch rate of highly doped p-type Si is the lowest compared to moderately and lightly doped p-type Si.^20^ Here, the Schottky barrier at the metal–semiconductor interface is suggested to play a crucial role. In the oxidative and reductive half-reactions, holes are injected into the valence band of Si. Due to the Schottky barrier interface between Au and Si, holes are confined at the interface for n-type Si, promoting surface oxidation and etching, whereas holes are driven away from the interface for p-type Si, which decelerates the oxidation and etching.^20^ Therefore, the etch rate of p-type Si is lower than n-type Si. In the case of MAPE, the inhibition of catalytic etching occurs at high dopant concentrations in both n- and p-type Si, where the Coulombic forces caused by Fermi-level pinning would be expected to be opposite in polarity. Therefore, the theory suggested for MACE does not sufficiently explain why the same inhibition is observed for both dopant types during MAPE. This suggests that the inhibition effect in highly doped MAPE-etched substrates is not a Fermi-level effect and proceeds by a different mechanism than in MACE.

The specific MAPE inhibition exhibited in the highly doped Si studies raises two intriguing questions: why does the inhibition happen only at high dopant concentrations, and why does it disappear as the etch progresses? The time dependence of the inhibition suggests that the effect happens due to an inhibitory effect only at the surface of the Si substrate, and as the general physicochemical RIE etch progresses and more of the top layers of Si are etched away, the inhibition diminishes. One possible explanation of this surface effect is the enrichment of the dopant atoms at the surface of the Si during the manufacturing or nanoparticle fabrication process, which impedes MAPE-enhanced etching. Enrichment of the surface region with dopants could be driven by lower energy of the dopant at the surface *versus* the bulk due to lattice relaxation or surface reconstruction. For instance, prior research shows that in Si(100) heavily doped with P (∼10^20^ atoms per cm^3^), dopant atoms are enriched at the surface compared to the bulk and can drive the thickening of the native oxide layer, even at room temperature.^32^ In addition, there is a pile-up of P at SiO_2_/Si interfaces, which increases with oxide thickness^33^ and is present with native oxides.^34^ The case is similar for p-type Si, with heavy doping with B resulting in subsurface B segregation and, for Si(100), a (2 × 1) surface reconstruction.^35^ Furthermore, the surface enrichment of dopants is well-known in silicon nanowires with a high surface-to-volume ratio.^36–38^ These phenomena may be enhanced during the nanofabrication processes, where techniques such as metal evaporation can induce substrate heating,^39,40^ promoting enhanced diffusion of the dopant atoms from the bulk to the surface.

A second possible mechanism is the formation of inhibitory chemistries at the surface of highly doped Si that may inhibit MAPE etching. In heavily doped polySi, the preferential formation of SiO_2_ layers at the surface in high oxygen concentrations inhibits bromine-based reactive plasma etching and causes a decreased etch rate in heavily doped polySi as compared to undoped polySi.^17^ The formation of borosilicate glass on the surface of heavily B-doped Si (B concentrations >10^19^ cm^−3^) is employed as an etch-stop barrier layer for alkaline, anisotropic wet etchants.^41–44^ It is proposed that the degeneration of the space charge layer on the Si surface above these B concentrations creates a potential well.^42^ Here, injected electrons from oxidation reactions rapidly combine with holes in the valence band, and the lack of surface electrons impedes the surface reactions necessary for subsequent etching. Similar inhibitory chemistries may be forming on the surface of the heavily doped Si, preventing the MAPE-enhanced etching until the physical plasma removes the surface layers.

Still, these mechanisms have unique features and chemistries depending on whether the dopant is supravalent or subvalent. However, no difference is observed across heavy doping with B, P, As, or Sb, suggesting that there is likely an additional mechanism at play that either is dominant or at least bridges any features that differ between the dopant atoms. Given that some differences will persist for any substrate-based mechanisms, the question arises: What role does the catalytic gold play that may give rise to the unique feature that all the highly doped substrates examined here can inhibit MAPE?

### Role of catalytic metal during MAPE

**Fig. 3:**
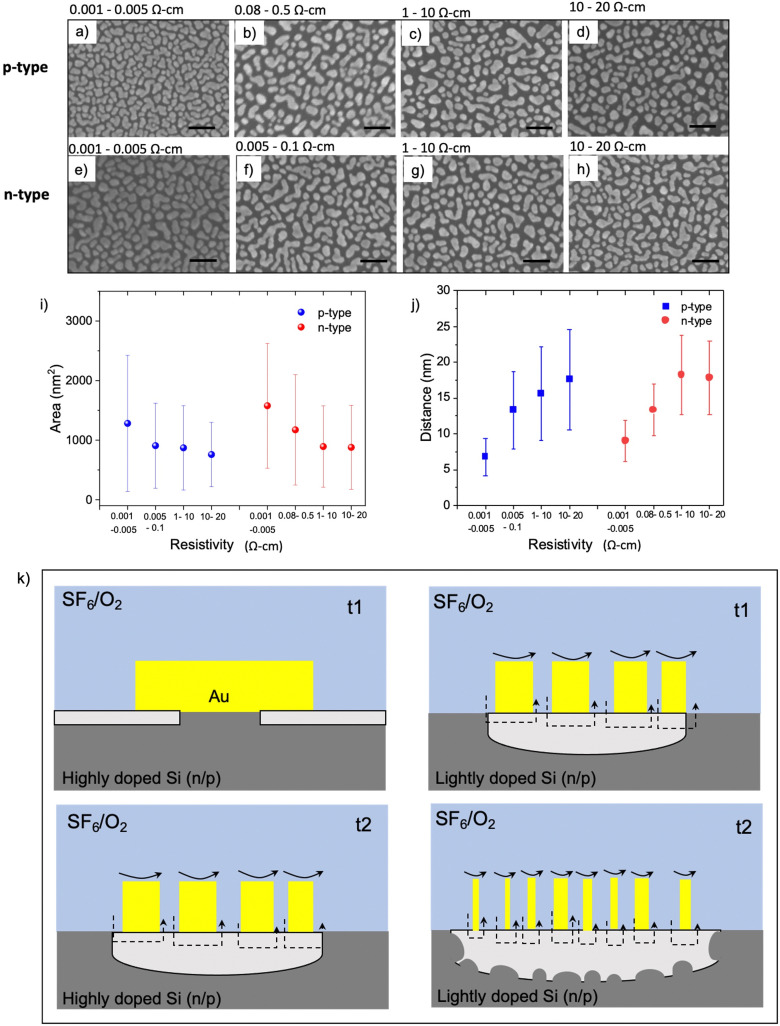
Au morphology on Si substrates with various resistivity ranges. (a–d) SEM images of 5 nm Au deposited by e-beam evaporation on p- and n-type silicon (e–h). All scale bars are 100 nm. (i) Average area of Au nanoparticles and islands and their interspacing, (j) as a function of resistivity for n- and p-type Si. (k) Schematic diagram showing the MAPE progression in highly doped Si (left) and lightly doped Si (right). For the lightly doped sample, there is more chemisorption of metal fluorides and other oxidising agents onto surfaces of smaller Au nanoparticles shown in black arrows, making Au particles more catalytically active and enhancing Si etching at the initial interval t1. As etching proceeds (t2), this catalytic effect is more prominent. However, in highly doped samples, the larger Au islands are formed, inhibiting the catalytic activity at the initial state (t1). As etching progresses to t2, the catalytic etching initiates after the Au nanoparticle sizes are reduced, resulting in the enhanced etching profile similar to the lightly doped substrate at t1.

It is well known that catalytic metals in various chemical reactions depend on the catalytic particle's size, shape, and morphology.^45,46^ Given that interfacial contact between the catalytic Au and Si is required for MAPE, we next investigated the morphology of 5 nm thick Au films deposited on various Si substrates using SEM. The size of nanoparticles and islands and their separation distance change for substrates with different doping levels. On highly n- and p-doped Si substrates, Au nanoparticles have coalesced and formed dense islands without reaching full coverage (Fig. 3a and e). When the doping concentration decreases to a moderate level, small isolated Au nanoparticles appear together among islands (Fig. 3b and f). Small nanoparticles dominate the lightly n- and p-type samples, as shown in Fig. 3c, d, g and h. The area of nanoparticles/islands and their distance for each resistivity range are presented in Fig. 3i and j, respectively. The average area of Au islands in highly doped n- and p-Si is 1200–1500 nm^2^. It decreases to 800–1000 nm^2^ and 700–800 nm^2^ for the moderately and lightly doped samples, respectively. The separation between nanoparticles and islands also exhibits similar trend for both n- and p-type samples, which is inversely proportional to resistivity.

Interestingly, the morphology and distribution of Au nanoparticles evaporated on Si with various doping levels show a similar tendency for both n- and p-type samples. This commonality likely arises from the enrichment of the surface with dopant atoms giving rise to different surface chemistries and structures that modulate the interfacial energy. In Si, heavy doping and surface enrichment with both n- and p-type dopants can lead to reconstructions that reduce the associated surface energy through the formation of new chemistries, a reduction in dangling bonds, and/or mitigating dopant-induced lattice strain. In doing so, these changes in interfacial energies can lead to differing driving forces for the spontaneous dewetting of Au on Si. The data in Fig. 3 suggest that the difference between the Au–Si and Si–air interfacial energies for highly doped silicon is lower than for lightly doped silicon, reducing the driving force underpinning dewetting. This dewetting process is likely occurring during the metal deposition process, given that thin evaporated metal films can reach 400–500 °C,^39^ surpassing both the semi-empirical Hüttig (128 °C) and Tamman (395 °C) temperatures for roughly estimating when surface and bulk atoms become mobile, respectively. Since this dewetting is happening only during or shortly after deposition likely limits the process to a couple of minutes, leading to partially dewetted films observed here in contrast with the more spherical particles obtained following annealing.^47^

During the process of MAPE, the geometry of the catalytic metal film is likely altered. In the case of Au, this is likely due to both the interfacial energy between Au and Si and sputtering due to the physical plasma etch, and may help explain the spreading and more chaotic etching during longer MAPE etches (*e.g.* 3 min). During RIE *via* SF_6_/O_2_ plasma, Au can be consumed through the formation of metal-fluorides and oxygen radicals such as AuF^+^, AuO^+^, and other reactive species.^48,49^ Many gold fluorides, such as AuF_5_, are strong oxidising agents and highly reactive,^50^ although due to the requirement for interfacial contact between the Au and Si for MAPE, if they are forming and facilitating MAPE, they need to be at the Au interface.^9^ Previous research has also shown that small Au nanoparticles between 3–5 nm are highly reactive in various chemical reactions, including CO oxidation,^45,51^ aerobic oxidation of alcohols,^52^ and hydrogenation.^53^ Specifically, the catalytic activity decreases significantly with increasing Au particle size.^45^ Density functional calculations show that small Au nanoparticles possess a larger number of low-coordinated atoms on the corners and the edges of nanoparticles than their surface, which act as active sites.^45^ These Au atoms are catalytic when they bind with the reactants, and increased catalytic activity with decreasing coordination numbers has been confirmed by several experimental observations.^54–56^

In our previous work,^9^ we proposed that the diffusion of metal ions through Si and the diffusion of Si into the Au surface occurring at the metal–Si interface causes oxidation of the underlying Si in the vicinity of metallic catalyst. In addition, metal-fluoride and other oxygen radicals, generated in SF_6_/O_2_ plasma, can chemisorb onto Au nanoparticles and create active catalytic sites leading to further oxidation of the underlying Si. Because catalytic activity depends on the size of Au nanoparticles,^45^ a prerequisite of the MAPE process may be reducing the metal film down to the regime that establishes catalytic activity. In highly doped n- and p-type substrates, spontaneously formed islands of Au following evaporation are the largest among other doping levels (Fig. 3a–h), resulting in lower overall surface area and less interaction between plasma and Au surface. Therefore, the chemisorption of metal fluorides and other oxidising agents on Au surfaces of the large nanoparticles, depicted in black arrows, is possibly much less than that on smaller Au nanoparticles (Fig. 3k). As a result, catalytic etching may be inhibited in the highly doped n- and p-type substrates at the early stage (t1), and the Si underlying the Au nanoparticles is only etched isotopically in the plasma, generating Si pillar features underneath each Au nanoparticle (Fig. 3k, left panel). Larger Au islands found on highly doped substrates require more time for plasma to interact with Au islands and etch them into smaller particles, eventually acting as a catalyst and enhancing the etching at the later stage (t2). This is evidenced in enhanced etching observed in both highly doped n- and p-type Si after 2–3 min of etching (Fig. S1 and S2, ESI†). Fig. 3k (right panel) suggests a mechanism relying on the smaller size of Au nanoparticles formed on a lightly doped Si being more catalytically active and generating enhanced etching. As etching continues, the catalytic activity becomes stronger owing to the reduction in the size of the Au nanoparticles. This increase in catalytic activity leads to a deeper etch into the Si surface and the expansion of etch pits and roughness caused by increased random movement of Au nanoparticles.

**Fig. 4:**
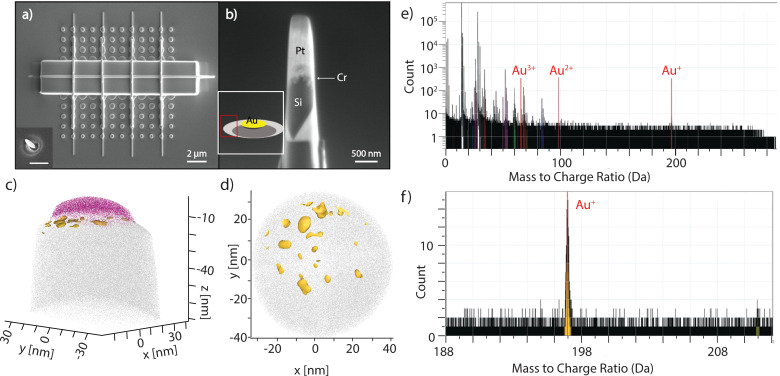
Atomic probe tomography analysis of MAPE samples. (a) A top view SEM image of nanoparticle array patterned onto n-type Si substrates (resistivity 0.08–0.5 Ω cm) after etching for 1 min. The sample was then coated with Cr and Pt capping layers by electron beam and FIB deposition. The inset of Fig. 4a is representative of sample (a) before coating. Scale bar is 500 nm. (b) An example of an APT specimen after lift-out and FIB milling. The inset of Fig. 4b shows a schematic of the etched structure for 1 min, and the peak region is labelled in red. (c) APT analysis performed on the specimen obtained from the sidewalls of the etched structure in (a). Au (yellow) was observed primarily at the interface of Cr (pink) and Si (gray) and found to be in small particles and clusters instead of as a homogenous layer. (d) Top-down view showing the Au was found as clusters and particles near the surface of the Si. (e) Whole mass spectra with Au species labeled. (f) Mass spectrum of the Au^+^ peak; under 2000 Au^+^ ions were detected in this specimen.

This model would suggest that small catalytic metal particles are forming in the regions of enhanced etching. Our previous study of MAPE on Si suggested that the Au metal acts as a catalyst and not a reagent in the Si etching reaction, and the consumption of Au happened not as part of the Si etching reaction but through secondary reactions instead.^9^ Tracking the movement of the Au metal as MAPE advances should provide important insights into Au diffusion into the Si due to catalyst activity. Atom Probe Tomography (APT) can provide a 3D atom-by-atom reconstruction of a nanoscale sample, making it a promising technique for characterising the Au catalyst in MAPE. Patterned n-type Si substrates with resistivity 0.08–0.5 Ω cm etched for 1 min under the same conditions shown in Fig. 1 were utilised for APT. This moderately doped sample is selected because it is in an early stage of MAPE initiation. Fig. 4a shows a corresponding etched single array with Pt lines and a main protective Pt layer. The inset of Fig. 4a is representative of the 1 min etched structure before coating. FIB lift-out and FIB sharpening^25^ were performed on selected regions to obtain APT specimens. Specifically, the APT specimens were fabricated from the centres of the etched pits (troughs) and areas originally not covered by nanoparticles (peaks). The schematic of the etched structure is shown in the inset of Fig. 4b, where the peak region is labelled in red, away from the original position of the fabricated Au nanoparticles. Fig. 4b shows an APT sample just prior to the final sharpening and the image shows the different layers of the structure. This is then sharpened to a diameter less than 100 nm and has the apex placed at the interface between the Cr and Si layers of the region of interest.

Data reconstruction of the analysed volume was carried out on AP Suite 6 (Cameca) using a voltage-based reconstruction protocol, with the parameters adjusted to match the plane spacings of the (100) silicon substrate. For specimens created from the peak region, Au^+^ ions were clearly seen at 197 Da as shown in Fig. 4e and f and they were found only at the very apex of the analysed volume immediately between the Cr capping layer and the Si substrate (Fig. 4c). It is clearly seen that Au was found to have coalesced into small clusters on the surface (Fig. 4d). The sizes of these Au clusters are in the few nm range, which agrees with the size of catalytically active Au.^45^ However, the exact size and shape of the clusters are challenging to quantify as they are affected by reconstruction artefacts due to the large evaporation field difference between Au and Si. No detectable levels of Au exist below this initial surface region. This data suggests the movement of Au away from the original interface of the nanoparticles and Si, which may be due to localised sputtering. Au is not detectable for the trough region, but the minimal protection from the physical etching of the plasma complicates the visualisation of Au in this area. The movement of Au away from the initial location as etching proceeds continues after 2 min of etching, with Au also found on the peak region (Fig. S4, ESI†).

More spreading of the remaining small Au nanoparticles with etch time suggests that Au nanoparticles are dispersed and consumed as part of the physical process of MAPE, and these catalytic Au particles create Au–Si contacts that undergo a catalytic reaction. Overall, this atomistic analysis confirms Au migration away from the starting position as etching proceeds, aligning with the expansion of the etch region with increasing etch time and further supporting the role of Au as a mobile catalyst consumed during the physical etching process. This suggests that methods to direct the movement of Au during etching and protect Au from physical etching may provide a pathway to controlling and extending the MAPE process.

By combining aspects of the mechanisms covered so far, it is possible to shape a hypothesis for how both highly doped n- and p-type Si inhibit MAPE. The first commonality discussed between highly doped n- and p-type Si is that the surface enrichment of dopant atoms can lead to chemical and structural changes. Interestingly, Au can serve as a substitutional atom in Si as a neutral, positive, or negative ion and generally diffuses through a ‘kick-out’ mechanism, with a non-negligible contribution from the vacancy-mediated Frank–Turnbull mechanism, especially at lower temperatures.^57^ This feature allows it to adapt to the type of dopant present, and past research shows that both highly doped n- and p-type Si increase the diffusion rate of Au into Si, albeit with B approximately an order of magnitude less efficient than P.^57^ The increase in diffusion can be multifaceted, with both ion pairing to dopant atoms and using a higher level of defects, such as vacancies and dislocations, leading to an increase in diffusivity. In fact, high levels of dopant atoms can give rise to the formation of new compounds, such as Au_2_P_3_^58^ and boron-silicate compounds,^59^ along with dislocations, which can serve as gettering sinks for Au atoms. This increase in diffusivity and gettering by these chemistries, along with the larger Au island sizes (Fig. 3), may lead to a relative deficit of the very small but highly catalytic Au nanoparticles on the highly doped n- and p-type wafers. However, once the process of plasma etching forms new, small catalytic Au nanoparticles while also removing any inhibitory surface chemistries, MAPE can then proceed, similar to the lightly doped substrates.

## 4. Conclusion

We have systematically explored the role of doping on MAPE, including the effects of the Si wafer dopant concentration and atom type. Interestingly, at the highest dopant concentrations, both n- and p-type Si initially exhibits inhibition of the MAPE-enhanced etching, in contrast with MACE. As the etch progresses, the etching enhancement, observed in the underlying Si at the metal–Si interface, results from the catalytic activity of nanometer-sized Au nanoparticles. We also provide the evidence *via* APT of Au on the Si surface, which is driven away from the original patterned location. It is hypothesised that the inhibition of MAPE is due to surface enrichment of the dopant atoms, the formation of different surface chemistries, and the morphology of Au nanoparticles. This study sheds further light on the unique MAPE mechanism driven by Au catalysts. Going forward, computational modelling may provide new insights into the interplay of the different mechanisms, helping to elucidate how they work together to facilitate MAPE for various dopant levels. Combined with the experimental data, these new insights may help solidify our understanding of this unique process. We expect that, together, this improved understanding will help optimise the parameters of catalytic etching in MAPE, and in turn, provide new possibilities for harnessing MAPE as a controllable dry etching process.

## Author contributions

Conceptualisation: JBS, BDA; methodology: JBS, NP, JOD, BDA; investigation: JBS, NP, JOD; formal analysis: JBS, NP, JOD; writing – original draft: JBS; writing – review & editing: JBS, NP, JOD, BDA; supervision: BDA.

## Conflicts of interest

There are no conflicts to declare.

## Supplementary Material

MH-010-D3MH00649B-s001
