# Supplementary material for: Doping density, not valency, influences catalytic metal-assisted plasma etching of silicon
Source: Mater Horiz. 2023 Jun 19;10(9):3393–403. doi: 10.1039/d3mh00649b (PMC10463556; doi:10.1039/d3mh00649b)
Supplement: MH-010-D3MH00649B-s001 [file MH-010-D3MH00649B-s001.pdf]

Electronic Supplementary Material (ESI) for Materials Horizons.
This journal is © The Royal Society of Chemistry 2023

Supplementary Information

Doping Density, Not Valency, Influences Catalytic Metal-Assisted Plasma Etching of Silicon

Julia B. Sun[1], Namphung Peimyoo[1], Douglas, James O[2] and Benjamin D. Almquist[1*]

[1]Department of Bioengineering, Imperial College London, Royal School of Mines, Exhibition Road, London SW7 2AZ, UK
[2]Department of Materials, Imperial College London, Royal School of Mines, Exhibition Road, London, SW7 2AZ, UK

*Corresponding author email: b.almquist@imperial.ac.uk

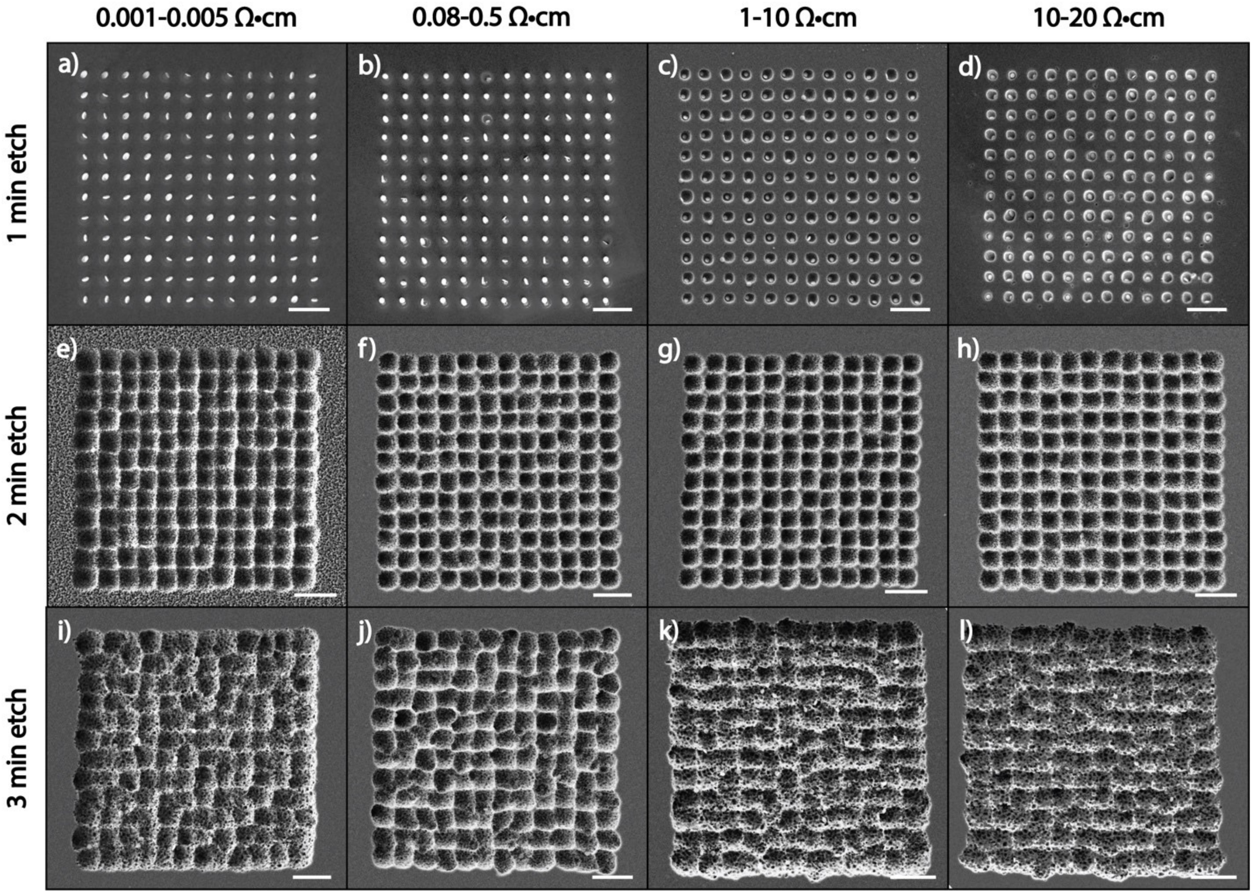

Figure S1. MAPE enhanced etching of n-type Si substrates. Circular nanoparticles with 5 nm Au and 10 nm Cr were patterned by electron beam lithography and metal deposition on n-type phosphorus doped Si substrates of varying levels of doping (resistivities 0.001-0.005, 0.08-0.5, 1-10, and 10-20 Ω-cm). The circular nanoparticles measured 200 nm in diameter and were patterned in 12x12 arrays. The level of doping decreases from the heaviest doped substrate in the leftmost column (resistivity 0.001-0.005 Ω-cm) to the lightest doped substrate in the rightmost column (resistivity 10-20 Ω-cm). Si substrates were etched for 1 min (top row), 2 min (middle row), or 3 min (bottom row) in mixed SF_6/O_2 plasma at 25% O_2 concentration. At 1 min, inhibition of the MAPE enhanced etching occurred in the heaviest doped Si substrates (resistivities 0.001-0.005 and 0.08-0.5 Ω-cm), which was visualized by the formation of Si pillars instead of etched pits underneath each Au/Cr nanoparticle. No inhibition of MAPE was seen in the lighter doped substrates (resistivities 1-10 and 10-20 Ω-cm) at 1 min etch. This inhibition effect was not seen in the n-type Si substrates at 2 and 3 min as MAPE appeared to proceed as expected. At 2 min, all substrates, regardless of doping level, exhibited etch pits characteristic of locally enhanced etching underneath each nanoparticle of the array. These etch pits became more irregular in border and shape as the etch advanced to 3 min of etching. Images for 1 min etches were taken at 45° tilt to better visualize the etch features. All scale bars represent 2 µm.

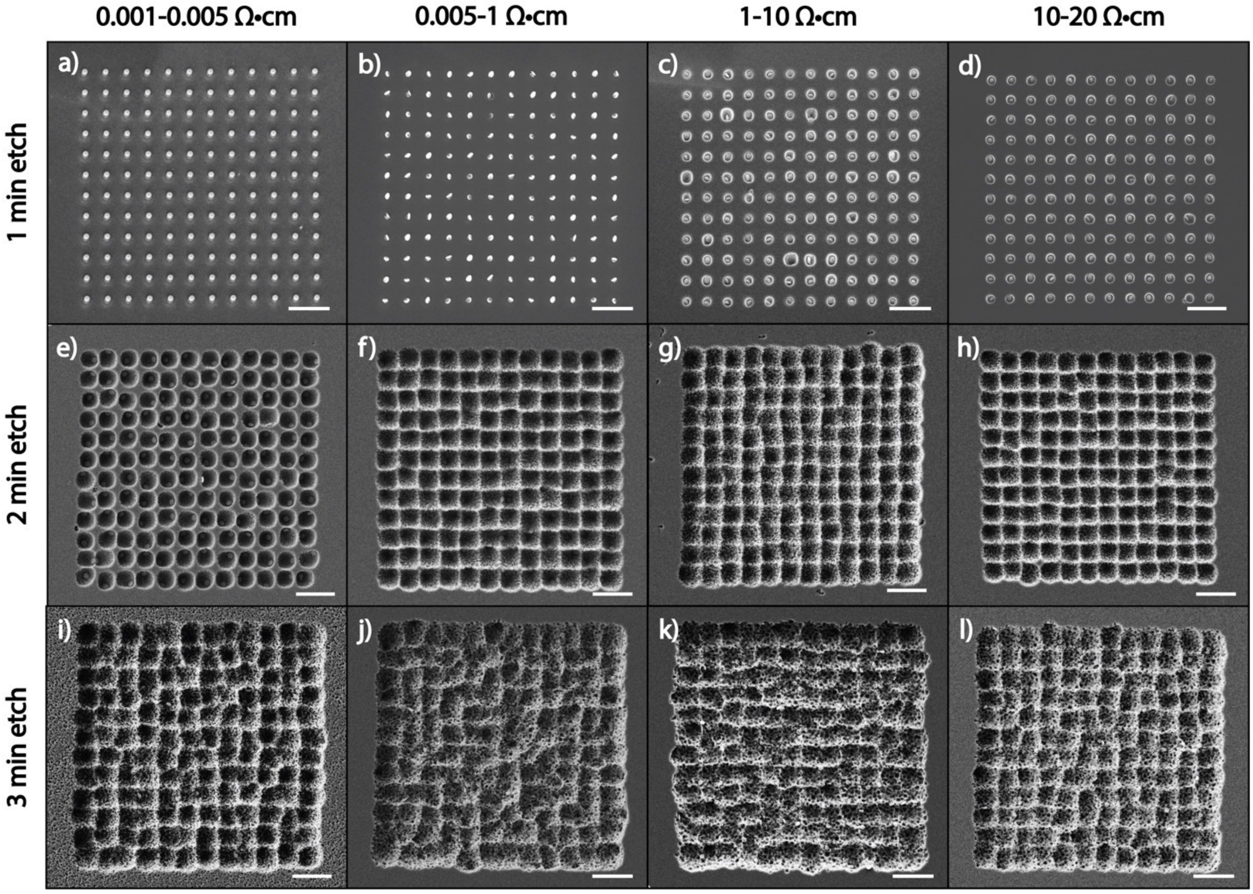

Figure S2. MAPE enhanced etching of p-type Si substrates. P-type boron-doped Si substrates with different levels of doping (resistivities 0.001-0.005, 0.005-1, 1-10, and 10-20 Ω-cm) were patterned with 12x12 arrays of circular nanoparticle by electron beam lithography. The nanoparticles measured 200 nm in diameter and were deposited with thin films of 5 nm Au and 10 nm Cr. The level of doping decreases from the heaviest doped substrate in the leftmost column (resistivity 0.001-0.005 Ω-cm) to the lightest doped substrate in the rightmost column (resistivity 10-20 Ω-cm). Si substrates were etched for 1 min (top row), 2 min (middle row), or 3 min (bottom row) in mixed SF_6/O_2 plasma at 25% O_2 concentration. Similar to the n-type Si, early inhibition of MAPE in the heavily doped substrates (resistivities 0.001-0.005 and 0.005-1 Ω-cm) was evident from the formation of Si pillar instead of etched pits at 1 min. This inhibition did not occur in the lightly doped substrates (resistivities 1-10 and 10-20 Ω-cm). By 2 and 3 min, all substrates exhibited MAPE enhanced etching as etch pits formed underneath each nanoparticle. No enhancement of etching occurred outside the boundaries of the original array even as the borders of the etch pits became more irregular as the etch proceeded. Images for 1 min etches were taken at 45° tilt. All scale bars represent 2 µm.

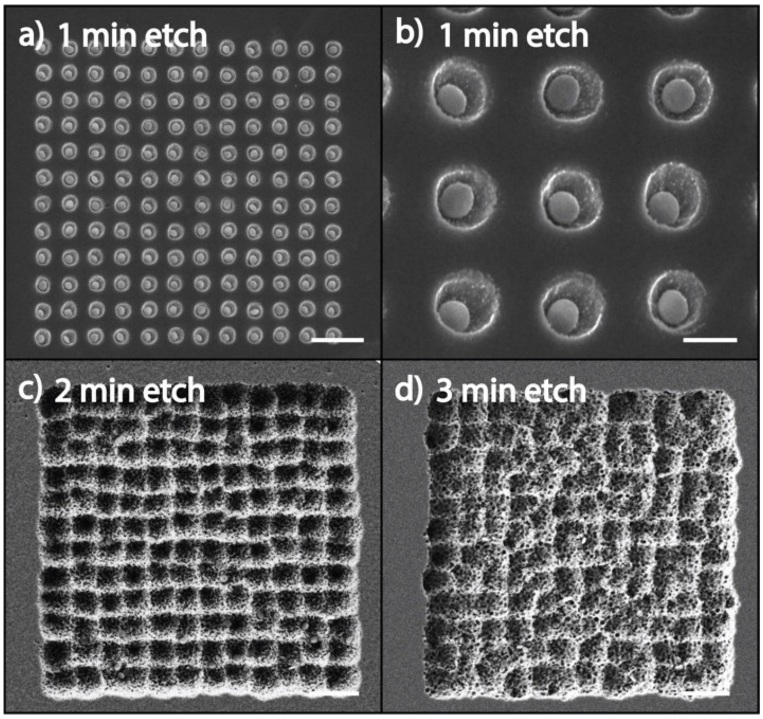

Figure S3. MAPE etching of undoped Si substrates. Undoped Si substrates were patterned with circular nanoparticles of 5 nm Au/10 nm Cr in 12x12 arrays and etched in SF_6/O_2 mixed plasma at 25% O_2 concentration. Panels a-b show tilted SEM images of etching at 1 min, panel c shows etching at 2 min, and panel d displays etching at 3 min. All undoped substrates exhibited MAPE-catalyzed etching at the Au-Si interface and no inhibition of MAPE. Scale bars for panels a, c, d represent 2 μm, and the scale bar for panel b represents 500 nm.

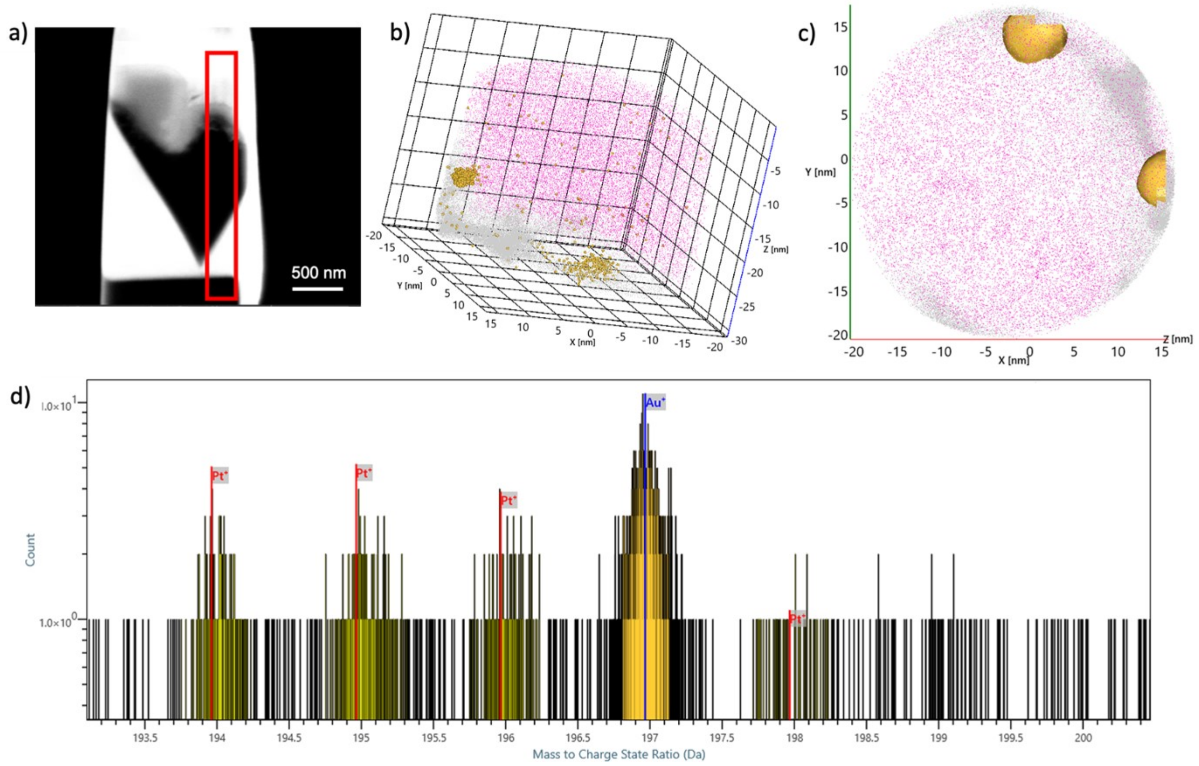

Figure S4. APT analysis of the 2 min etched structure found in the n-type Si substrate. (a) APT specimen collected from the edge of the etch pits (peak region). (b) APT analysis obtained from the region marked in (a). Au (yellow) was observed at the interface of Cr (pink) and Si (gray). (c) Top-down view showing Au was found as clusters and particles near the surface of the Si. The characteristics of small Au particles are similar to what observed in 1 min etch sample, but they are found further away from the original position of the as prepared nanoparticle. (d) Mass spectrum of the Au^+ peak; under 650 Au^+ ions were detected in this sample.
